# Cell-Free DNA (cfDNA) Regulates Metabolic Remodeling in the ES-2 Ovarian Carcinoma Cell Line, Influencing Cell Proliferation, Quiescence, and Chemoresistance in a Cell-of-Origin-Specific Manner

**DOI:** 10.3390/metabo15040244

**Published:** 2025-04-02

**Authors:** Isabel Lemos, Catarina Freitas-Dias, Ana Hipólito, José Ramalho, Fabrizio Carteni, Luís G. Gonçalves, Stefano Mazzoleni, Jacinta Serpa

**Affiliations:** 1 iNOVA4Health, NOVA Medical School, Faculdade de Ciências Médicas, Universidade NOVA de Lisboa, Campo dos Mártires da Pátria, 130, 1169-056 Lisbon, Portugal; a2022507@nms.unl.pt (I.L.); catarina.freitasdias@nms.unl.pt (C.F.-D.); ana.hipolito@nms.unl.pt (A.H.); jose.ramalho@nms.unl.pt (J.R.); 2 Instituto Português de Oncologia de Lisboa Francisco Gentil (IPOLFG), Rua Prof Lima Basto, 1099-023 Lisbon, Portugal; 3 Lab Applied Ecology and System Dynamics, Dipartimento di Agraria, Università di Napoli “Federico II”, Portici, 80055 Naples, Italy; fabrizio.carteni@unina.it (F.C.); stefano.mazzoleni@unina.it (S.M.); 4 Instituto de Tecnologia Química e Biológica António Xavier (ITQB NOVA), Avenida da República (EAN), 2780-157 Oeiras, Portugal; lgafeira@itqb.unl.pt

**Keywords:** cfDNA, metabolic remodeling, cell proliferation, quiescence, migration, chemoresistance, ovarian clear cell carcinoma

## Abstract

**Background:** The cell-free DNA (cfDNA) is an extracellular fragmented DNA found in body fluids in physiological and pathophysiological contexts. In cancer, cfDNA has been pointed out as a marker for disease diagnosis, staging, and prognosis; however, little is known about its biological role. **Methods:** The role of cfDNA released by ES-2 ovarian cancer cells was investigated, along with the impact of glucose bioavailability and culture duration in the cfDNA-induced phenotype. The effect of cfDNA on ES-2 cell proliferation was evaluated by proliferation curves, and cell migration was assessed through wound healing. We explored the impact of different cfDNA variants on ES-2 cells’ metabolic profile using nuclear magnetic resonance (NMR) spectroscopy and cisplatin resistance through flow cytometry. Moreover, we assessed the protein levels of DNA-sensitive Toll-like receptor 9 (TLR9) by immunofluorescence and its colocalization with lysosome-associated membrane protein 1 (LAMP1). **Results:** This study demonstrated that despite inducing similar effects, different variants of cfDNA promote different effects on cells derived from the ES-2 cell line. We observed instant reactions of adopting the metabolic profile that brings back the cell functioning of more favorable culture conditions supporting proliferation and resembling the cell of origin of the cfDNA variant, as observed in unselected ES-2 cells. However, as a long-term selective factor, certain cfDNA variants induced quiescence that favors the chemoresistance of a subset of cancer cells. **Conclusions:** Therefore, different tumoral microenvironments may generate cfDNA variants that will impact cancer cells differently, orchestrating the disease fate.

## 1. Introduction

Liquid biopsies are minimally invasive approaches for obtaining tumor-derived genetic material, such as DNA, circulating tumor cells, and extracellular vesicles, which can provide molecular features about the tumor and its microenvironment [1,2,3]. The presence of fragmented extracellular double-stranded DNA, the so-called cell-free DNA (cfDNA), in human peripheral blood was firstly reported by Mandel and Metais in 1948 [4]. However, the precise roles of cfDNA and the mechanisms controlling its release into the blood stream have not yet been established. Studies have proposed different phenomena allowing cfDNA release, such as the loss of cell membrane integrity, due to cellular injury or cell death, and following active and concerted secretion [5]. The fragmentation pattern, including length and sequence, can reflect the cfDNA tissue of origin and somehow indicate its release mechanisms [6,7]. Albeit elevated levels of cfDNA may result from non-malignant causes, it was reported that cfDNA levels were elevated in cancer patients compared to healthy individuals, stablishing a link between cfDNA and cancer [8,9,10]. In the recent years, cfDNA has been explored in the peripheral blood of cancer patients, as a potential biological source for the determination of biomarkers for diagnosis, prognosis, and follow-up [11].

In an era of personalized medicine, being able to target cancer-related mutations constitutes a fundamental part of oncology, since the released fragments retain the genetic and epigenetic signatures from the cell of origin [12], and the tumor cfDNA offers a source of cancer-related genetic alterations, as it is indicated as an important biomarker for cancer follow-up and management by different studies [13,14,15]. There are no doubts about the potential use of cfDNA as a powerful tool in decision making and oncological patients’ management; however, its biological relevance in tumor malignancy remains under explored. The cfDNA was already posited as an important promoter of cancer metastasis [16,17], but the exact mechanisms through which cfDNA regulates cancer cell features needed for disease progression, such as cancer metabolic remodeling, remain unclear.

Among gynecological malignancies, ovarian cancer corresponds to the second most prevalent malignancy and the leading cause of mortality [18]. Despite the development of more advanced therapies for ovarian cancer, the primary treatment remains pharmacotherapy, which typically is based on compounds from taxane family, and on platinum-based compounds [19]. The diagnosis at advanced stages and chemotherapy-resistance contributes to the poor prognosis of ovarian cancer [20,21]. Ovarian carcinoma (OC) is the most common type among ovarian malignancies, accounting for 90% of the cases [22,23], and it comprises different histological types, with thigh-grade ovarian serous carcinoma (OSC) being the most prevalent [21,24]. OSC is frequently diagnosed at an advanced stage and frequently acquires chemoresistance, as opposed to ovarian clear cell carcinoma (OCCC), which is an uncommon but highly mortal histotype presenting intrinsic chemoresistance [25,26]. Despite its low incidence, OCCC still represents a challenge in its clinical management as a biologically distinct entity, and it presents the worst prognosis owing to its innate chemoresistance [27,28]. Overcoming cisplatin resistance is critical to increasing survival rates and providing more effective, individualized cancer treatments. The presence of elevated levels of cfDNA in ascites and peripheral blood of ovarian-cancer patients was reported [29,30,31,32]. Our team extensively reported the metabolic remodeling and cysteine reliance as pivotal events for OCCC chemoresistance and aggressiveness [33,34].

CfDNA has considerable potential as a biomarker for ovarian cancer, allowing for non-invasive disease monitoring, selection of appropriate therapies, and insights into tumor heterogeneity and chemoresistance [35]. Low cfDNA concentrations, specifically in early disease stages; interference of non-tumoral DNA; and a lack of standardized protocols for collection, processing, and analysis, all pose challenges to its clinical applicability [36]. Furthermore, the biological relevance of cfDNA in tumor growth remains unknown, and our study intends to gain a deeper insight into this role. Herein, we explored if cfDNA released by ES-2 cells, an OCCC cell line, plays a role in the metabolic remodeling and in the promotion of chemoresistance. Moreover, and because glucose bioavailability interferes with living systems [37], we tested whether glucose bioavailability and culture duration generate cfDNA that could impact the phenotypic features and the metabolic profiles of ES-2 cells differently.

## 2. Materials and Methods

### 2.1. Cell Culture

Ovarian clear cell carcinoma cell line ES-2 (ATCC^®^ CRL-1978™) was obtained from American Type Culture Collection (ATCC).

Cells were maintained at 37 °C in a humidified 5% CO_2_ atmosphere and cultured in Dulbecco’s Modified Eagles’ Medium (DMEM; 41965-039, Gibco, Life Technologies, Waltham, MA, USA) supplemented with 10% fetal bovine serum (FBS; S0615, Merck, Rahway, NJ, USA), 1% antibiotic–antimycotic (AA; P06-07300, PAN Biotech, Aidenbach, Germany), and 50 µg/mL Gentamicin (15750-060, Gibco, Life Technologies, MA, USA). Cells were cultured to approximately 75–100% of optical confluence before they were detached with 0.05% trypsin–ethylenediaminetetraacetic acid (EDTA) 1× (25300-054, Invitrogen, Waltham, MA, USA).

### 2.2. Genomic Material Extraction

Cells were plated in T-75 flasks and grown to ~80% confluency in supplemented DMEM. For DNA extraction from cell culture conditioned media, cells were cultured in FBS-free culture medium in the absence or presence of glucose (5 mM) for 6 and 48 h. DNA extraction was performed using QIAamp^®^ DNA Blood Mini Kit (51106, Qiagen, Hilden, Germany), according to the manufacture’s recommendations. Four different variants of cfDNA were obtained based on the glucose bioavailability and the time of incubation: Early-cfDNA-Gluc, cfDNA isolated from cells that were in the presence of glucose for 6 h; Early-cfDNA-NoGluc, cfDNA isolated from cells that were in the absence of glucose for 6 h; Late-cfDNA-Gluc, cfDNA isolated from cells that were in the presence of glucose for 48 h; and Late-cfDNA-NoGluc, cfDNA isolated from cells that were in the absence of glucose for 48 h. All the DNA material used in this work was aliquoted and stored at −20 °C until further use.

### 2.3. Cell Proliferation Assessment

For cell proliferation analysis, cells were seeded in 96-well plates (1 × 10^4^ cells/well) in complete DMEM, synchronized under starvation, and exposed to the experimental conditions: 10 ng/mL of the 4 different variants of cfDNA in the presence (5 mM) or absence of glucose. At 6 or 48 h of exposure, cells in suspension were collected and adherent cells were harvested, added to the supernatants and centrifuged at 155× *g* for 5 min. Supernatants were discarded and cells were counted with Trypan Blue Staining (15250-061, Gibco) using a Neubauer improved counting chamber.

### 2.4. Wound Healing Assay

The migration capacity upon cfDNA exposure was assessed by the wound healing assay. Cells were seeded in 12-well plates (2.5 × 10^5^ cells/well) in supplemented DMEM and maintained until 70–80% of confluence. Cells were synchronized under starvation and treated with Mitomycin-C (5 µg/mL, M4287, Sigma, MA, USA), and after 3 h, the cell monolayer was scratched with a 200 µL pipette tip to create a wound. Cells were washed in PBS 1× and exposed to control conditions and the variants corresponding to the late cfDNAs. The wound closure was monitored by photography at 0, 2, 6, 8, 10, 24, 28, 32, and 48 h using phase-contrast microscopy with the Olympus IX53 Inverted Microscope. Images were acquired and processed with Olympus cellSens v.1.17 software. ImageJ software v1.53e (https://imagej.nih.gov, downloaded on 1 October 2021) was used to analyze and quantify the images.

### 2.5. Nuclear Magnetic Resonance (NMR) Spectroscopy

Nuclear Magnetic Resonance (NMR) spectroscopy was performed to examine the exometabolome upon DNA exposure. Cells were cultured in T75 Flasks in glucose-free DMEM (P04-01549, Pan-Biotech), supplemented with 1% AA and either with or without 5 mM D-glucose, for 6 h and 48 h. Culture media was collected and stored at −80 °C.

New ES-2 cells were seeded in 24-well plates (1 × 10^5^ cells/well). Cells were synchronized under starvation and exposed to 5 mM D-glucose and to the 4 variants of cfDNA to a final volume of 600 µL for 48 h. Supernatants were collected and stored at −80 °C after being centrifuged for 2 min at 1200 rpm, and pellets were discarded.

To 540 µL of supernatants was added 30 µL of 0.4% (*v*/*v*) sodium azide in deuterated water (D_2_O) and 30 µL of a solution of 2.2 mM 3-(trimethylsilyl)propionic2,2,3,3-d4 acid (TSP) as an internal ^1^H NMR quantification and chemical shift reference. ^1^H NMR spectra were acquired at 25 °C in an UltrashieldTM Avance 500 Plus spectrometer (Bruker, Singapore) equipped with TCI-Z probe. A *noesypr1d* pulse program was used for the ^1^H-NMR (128 scans, relaxation delay 3 s, mixing time of 10 ms, spectral width of 11.7616 ppm, and free induction decay (FID) size of 65 k points). Spectra were acquired and processed using TopSpin 4.1 software (Bruker), and the assignments were made by resorting to spectral databases: Human Metabolome (HMDB) and Chenomx NMR Suite 8.11. The quantification of the identified metabolites was performed with Chenomx NMR Suite 8.11, using the TSP signal as the referenced concentration.

### 2.6. Cell Death Analysis

Flow cytometry was used to determine whether cfDNA had a protective impact against cell death. ES-2 cells were plated in 24-well plates (1 × 10^5^ cells/well) and cultured in 25 mM of glucose condition and stimulated with 10 ng/mL of the 4 different variants of cfDNA. After 24 h, cells were exposed to 0.402 mM L-cysteine (102839, Merck) and/or hypoxia induced with 0.1 mM CoCl_2_ (7646-79-9, Sigma-Aldrich, St. Louis, MI, USA), combined with 10 ng/mL of each variant of cfDNA. In addition, cells were exposed to the previous conditions combined with 25 µg/mL cisplatin for 24 h. After experimental conditions, conditioned culture media were collected, and adherent cells were harvested with trypsin; together with the supernatants, they were centrifuged at 150× *g* for 3 min. Following that, we performed incubation with 0.5 µL Annexin V-fluorescein (FITC)- (640906, BioLegend, San Diego, CA, USA) in 100 µL annexin V binding buffer 1 × at room temperature (RT), in the dark, for 15 min. After incubation, cells were rinsed with 200 µL PBS 1 ×—0.1% (*v*/*w*) BSA and centrifuged at 150× *g* for 2 min. Cells were resuspended in 100 µL of annexin V binding buffer 1×, and 1.25 µL propidium iodide (PI, 50 µg/mL; P4170, Sigma-Aldrich) was added. Acquisition was performed with BD AccuriTM C6 flow cytometer, Becton Dickinson, and data were analyzed using BD Accuri Software v1.0.34.1.

### 2.7. Immunofluorescence Analysis

Immunofluorescence was used to detect the expression of xCT protein to assess if it has association with chemoresistance [38]. Cells were seeded in 24-well plates (1 × 10^5^ cells/well) on glass coverslips with 0.2% gelatin coating from porcine skin (G-1890, Sigma-Aldrich) and cultured in 25 mM of glucose condition and stimulated with 10 ng/mL of the different variants of cfDNA. After 24 h, cells were exposed to 0.402 mM L-cysteine and/or hypoxia induced with 0.1 mM CoCl_2_ combined with 10 ng/mL of each variant of cfDNA. The expression of TLR9 was evaluated to follow the endocytosis related to cfDNA and eventual TLR9 turnover. This process was supported by the co-localization of TLR9 with LAMP1, a lysosomal marker indicating the digestion of TLR9-associated vesicles. The levels of p-NFkB and p-ERK1/2 were determined to assess the activation of TLR9-dependent signaling upon each cfDNA variant’s stimulation [39,40]. After experimental conditions, cells were fixed with 4% paraformaldehyde for 15 min at RT. Subsequently, cells were incubated with 50 mM ammonium chloride (NH_4_CL) for 10 min at 4 °C and permeabilized with saponin 0.1% in PBS 1×—0.5% BSA (*w*/*v*) for 15 min at RT. Cells were then incubated with rabbit anti-human xCT (1:500, ab37185, Abcam), rabbit anti-human p-NFкB (1:200, MA5-15160, Invitrogen), rabbit anti-human p-ERK1/2 (1:500, 9101, Cell Signaling, Danvers, MA, USA), and mouse anti-human TLR9 (1:200; MA5-38645, Invitrogen) overnight at 4 °C; and rabbit anti-human LAMP1 (1:200; MA5-29385, Invitrogen) for 3 h at RT. Cells were then incubated with secondary antibodies Alexa Fluor 488 anti-rabbit (1:1000; A-11034, Invitrogen), Alexa Fluor 488 anti-mouse (1:1000; 115-545-003, Invitrogen), and Alexa Fluor 594 anti-rabbit (1:1000; A-11037, Invitrogen) for 2 h at RT.

The slides were mounted in VECTASHIELD media with 4′-6-diamidino-2-phenylindole (DAPI) (Vector Labs, Newark, CA, USA) and examined by standard fluorescence microscopy under a Zeiss Imager.Z1 AX10 microscope. Images were acquired and processed with CytoVision v.7.1 software and quantified with ImageJ software, and in order to quantify the degree of colocalization between TLR9 and LAMP1, we used the JACoP tool.

### 2.8. Quantification of Cytoplasmic ROS by Flow Cytometry

To explore whether the protective role promoted by cfDNA variants was associated with the decrease in intracellular ROS levels, we determined its levels using 2′-7′-Dichlorodihydrofluorescein diacetate (DCFH-DA), which, when in contact with ROS, originates DCF, allowing its detection by flow cytometry [41]. ES-2 cells were plated in 24-well plates (1 × 10^5^ cells/well) and cultured in 25 mM of glucose condition and stimulated with 10 ng/mL of the 4 different variants of cfDNA. After 24 h, cells were exposed to 0.402 mM L-cysteine and/or hypoxia induced with 0.1 mM CoCl_2_ combined with 10 ng/mL of each variant of cfDNA. In addition, cells were exposed to the previous conditions and combined with 25 µg/mL cisplatin for 24 h. After experimental conditions, adherent cells were harvested with trypsin and centrifuged at 150× *g* for 3 min. Followed by incubation with the DCF-DA probe (2 µM, D6883, Sigma Aldrich) for 15 min at 37 °C, cells were resuspended in 10 µL of DMEM. Acquisition was performed with BD AccuriTM C6 flow cytometer, Becton Dickinson, and data were analyzed using BD Accuri Software v1.0.34.1.

### 2.9. Quantification of Lipid Peroxides by Flow Cytometry

Ferroptosis depends on the accumulation of lipid peroxides [42]. Therefore, to evaluate the effect of cfDNA variants on the levels of lipid peroxides, we performed a cytochemical staining with Bodipy 581/591 C11, as its fluorescence shifts from red to green upon oxidation by free radicals, allowing its detection by flow cytometry, providing a measurement of lipid peroxidation levels [43]. This assay was performed simultaneously with the ROS assay, allowing the association between ROS and lipid peroxides levels. Cells were plated in 24-well plates (1 × 10^5^ cells/well), and after experimental conditions, conditioned culture media were collected, and adherent cells were harvested with trypsin, and together with the supernatants, they were centrifuged at 150× *g* for 3 min. Following this, we performed incubation with Bodipy 581/591 C11 in 2% FBS—1× PBS (2 µM, D3861, Invitrogen) for 30 min at 37 °C. After incubation, cells were rinsed with 100 µL 2% FBS—1× PBS and centrifuged at 150× *g* for 2 min. Cells were resuspended in 200 µL 2% FBS—1× PBS, and data were acquired using the BD AccuriTM C6 flow cytometer, Becton Dickinson, and analyzed using BD Accuri Software v1.0.34.1.

### 2.10. Lentiviral Transduction

ES-2 cells expressing the green fluorescent protein (GFP) were generated by lentiviral transduction. ES-2 cells (1.5 × 10^5^/well) were seeded in 6-well plates and infected with Lv-GFP with 6 µg/mL polybrene (Hexadimethrine bromide, H9268, Sigma-Aldrich). Twenty-four hours after infection, media were replenished, and cells were selected with 1 µg/mL puromycin for 96 h. The efficiency of lentivirus infection was determined by GFP fluorescence intensity measured with the BD AccuriTM C6 flow cytometer, Becton Dickinson. Transduced ES-2 cells were then cultured in control conditions and in the presence of 10 ng/mL of each cfDNA variant for 10 days and for long-term exposure for at least 4 weeks.

### 2.11. Co-Culture of ES-2 and ES-2-GFP Cells

Co-cultures of ES-2 cells and ES-2-GFP cells previously selected by continuous exposure to the cfDNA variants were established directly using a ratio of 1:1 of ES-2 to ES-2-GFP cell number for 48 h and 72 h and exposed to the corresponding cfDNA variant. Competition assays were performed by flow cytometry, evaluating the number of cells that expressed GFP. Acquisition was performed with BD AccuriTM C6 flow cytometer, Becton Dickinson, and data were analyzed using BD Accuri Software v1.0.34.1. The assessment of the migratory capacity of cfDNA selected cells was also evaluated by wound-healing assay. The wound closure was monitored by photography at 0, 8, 24, and 32 h using the Olympus IX53 Inverted Microscope. Images were acquired and processed with Olympus cellSens software. ImageJ software v1.53e (https://imagej.nih.gov, downloaded on 1 October 2021) was used to analyze and quantify the images.

### 2.12. Analysis of Ki67 Marker by Flow Cytometry

On monocultures of each cfDNA, selected cells’ proliferation was assessed through evaluation of the expression of Ki67 proliferation marker by flow cytometry. Cells were collected and centrifuged at 150× *g* for 3 min, followed by incubation with primary antibody anti-Ki-67 clone SP6 (1:500; MA5-14520, Invitrogen) for 1 h. After incubation, cells were rinsed with PBS 1×—0.1% BSA (*w*/*v*) and centrifuged at 150× *g* for 2 min. After incubation with secondary antibody Alexa Fluor 594 anti-rabbit (1:1000; A-11037, Invitrogen) for 30 min, cells were resuspended in PBS 1×—0.1% BSA (*w*/*v*). Data were acquired using the BD AccuriTM C6 flow cytometer, Becton Dickinson, and analyzed using BD Accuri Software v1.0.34.1.

### 2.13. Quantitative Real-Time PCR (qPCR)

*CDKN1A* and *TP53* genes’ expression was analyzed by qPCR to assess the influence of cfDNA on the induction of a quiescent state, since the proteins encoded by these genes are important cell cycle regulators [44,45]. Total RNA was extracted using RNeasy Mini Extraction kit (74,104, Qiagen, Hilden, Germany), and cDNA was synthesized from 0.5 μg RNA by SuperScript II Reverse Transcriptase (18080e44, Invitrogen), both according to the manufacturer’s protocol.

Real-time PCR was carried out during 40 amplification cycles, according to manufacturer’s instructions, using a Lightcycler^®^ 480 System instrument (05015243001, Roche, Basel, Switzerland). The target genes were the following: *CDKN1A* (Fw: 5′-GAGACTCTCAGGGTCGAAAAC-3′; Rv: 5′-ATTAGGGCTTCCTCTTGGAGA-3′) and *TP53* (Fw: 5′-TGGGACAGCCAAGTCTGTGA-3′; Rv: 5′-CAGTTGGAAAACATCTTGTTG-3′). *Hypoxanthine-guanine phosphoribosyltransferase* (*HPRT*) was used as a housekeeping gene (Fw: 5′-TGACACTGGAAAACAATGCA-3; Rv: 5′-GGTCGTTTTTCACCAGCAAGCT-3′).

### 2.14. Generation of Spheroids

Co-cultures of ES-2 cells and ES-2-GFP cells previously selected by continuous exposure to the cfDNA variants were established directly using a ratio of 1:1 of ES-2 to ES-2-GFP cell number. A total number of 2 × 10^4^ cells/well were plated on a Nunclon Sphera 96-well plate (174925, Thermo Fisher, Waltham, MA, USA) and centrifuged at 290× *g* for 3 min. The next day, 100 µL of complete medium with 3 μg/mL collagen I (5409, PureCol, CA, USA) was added to each well and centrifuged at 100× *g* for 3 min. The next day, 100 µL of complete medium was added, and spheroid formation was allowed over the next 3–5 days.

### 2.15. Spheroid Sprouting

In vitro models of multicellular spheroids embedded in extracellular matrices (ECMs) are frequently used to study the mechanisms of cell invasion into ECM [46]; therefore, we assessed spheroid sprouting to evaluate how cfDNA affects invasive potential and metastatic behavior. The volume of each well was partially aspirated on top of the spheroid without disturbing it, until a final volume of 30 µL of complete medium. Then, 30 µL of Matrigel (354230, Corning, Corning, NY, USA), with or without each cfDNA variant, was added to each well, and the plate was incubated at 37 °C, in 5% CO_2_, for at least 2 h to allow Matrigel solidification with embedded spheroid. After Matrigel solidification, 100 µL of medium without serum supplemented with 0.5% of BSA was added to each well; the medium was refreshed every day. Spheroids were allowed to invade for 5 days. The spheroid sprouting was monitored by photography on days 1, 3, and 5, using phase-contrast microscopy and fluorescence microscopy with the Olympus IX53 Inverted Microscope. Images were acquired and processed with Olympus cellSens software. ImageJ software (https://imagej.nih.gov, downloaded on 1 October 2021) was used to analyze and quantify the images. The area of the spheroid core and the sprouting area were quantified.

### 2.16. Statistical Analysis

Statistical analysis was performed using GraphPad Prism 7 software (https://www.graphpad.com/, downloaded on 20 September 2020). Sample data were presented as the mean (normal distribution) ± SD. Assays were performed with three biological replicates per treatment (N = 3). Comparisons between data from each group were statistically analyzed by a two-tailed unpaired Student’s *t*-test, and multiple comparisons were performed using One-way ANOVA or two-way ANOVA with Dunnett’s or Tukey’s test, considering *p* < 0.05 as statistically significant. Multivariate statistical analysis of ^1^H-NMR data was performed on MetaboAnalyst 5.0 (assessed on 22 June 2024), using metabolites’ concentrations as inputs, and scaled using pareto-scaling. Heatmaps representing the univariate analysis of the extracellular levels of the different metabolites detected by NMR were created using MetaboAnalyst 5.0, and the parameters that were used for the analysis were the Euclidean distance measure and the Ward cluster algorithm.

## 3. Results

### 3.1. Glucose Availability Impacts the Exometabolome of Cell Subsets Cultured During 48 H

The impact of glucose bioavailability on ES-2 cells’ metabolic profile for 6 and 48 h of culture was evaluated in the exometabolome of cells, defined in the conditioned culture media by NMR spectroscopy.

**Figure 1:**
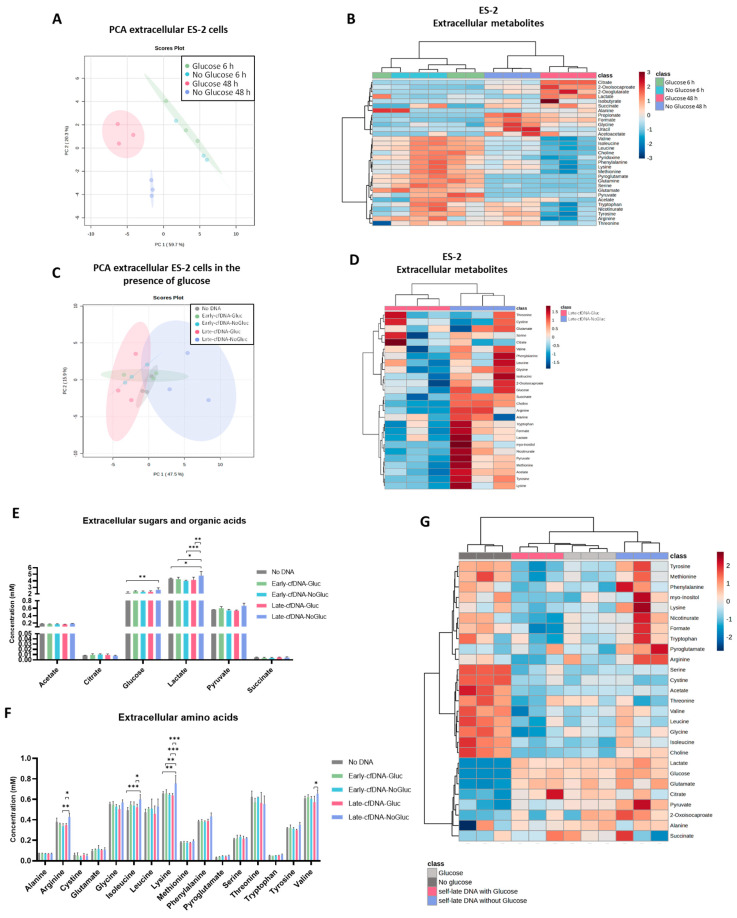
The Late-cfDNAs promote a metabolic remodeling in new ES-2 cells, resembling the metabolic profile of the cells of origin of these cfDNAs. Cells were cultured in the presence (5 mM) or absence of glucose for 6 h and 48 h, giving rise to 4 different variants of cfDNA. New ES-2 cells were exposed to each variant of cfDNA in the presence of glucose for 48 h. The exometabolome was analyzed by nucleal magnetic resonance (NMR). (**A**) Principal component analysis (PCA) score plot depicts the clustering pattern from the metabolic profiles concerning the bioavailability of glucose and the time of incubation. (**B**) Heatmap showing the levels of the metabolites identified on ES-2 supernatants. (**C**) PCA scores plot of metabolome data of ES-2 cells in control conditions and exposed to each variant of cfDNA. (**D**) Heatmap of the 28 identified extracellular metabolites on ES-2 upon stimulation with both late variants of cfDNA. (**E**) Levels of extracellular sugars and organic acids for 48 h of experimental conditions. (**F**) Extracellular amino acids’ levels on ES-2 upon 48 h of experimental conditions. (**G**) Heatmap showing the concentrations of extracellular metabolites in ES-2 cells treated with both late cfDNA variants and the condition media that originated them. The color code inside the heatmap depicts the relative fold change in each metabolite between classes: red and blue represent increased or decreased levels, respectively. Euclidean distance measure and the Ward cluster algorithm were the parameters used for the heatmap analysis. Results concerning the metabolite levels are shown as mean ± SD. * *p* < 0.05, ** *p* < 0.001 and *** *p* < 0.001. Two-way ANOVA was used, followed by Tukey’s test.

Principal component analysis (PCA) was performed using the 28 metabolites identified in conditioned media (Figure 1A). PCA score plot showed a clustering of the samples cultured for 6 h independent of the presence or absence of glucose. However, a clear separation was observed based on the culture duration between samples cultured for 6 h and 48 h. Moreover, at 48 h of culture, a separation was observed between samples cultured in the absence and presence of glucose, indicating that glucose, as a carbon source, influenced the exometabolome of ES-2 cells (Figure 1A).

The heatmap representing the univariate analysis of the metabolite’s levels identified on the supernatants confirmed the distinct metabolic profiles regarding glucose availability and highlighted that the presence of glucose increased the consumption of amino acids (Figure 1B).

### 3.2. The Late cfDNAs Promote a Metabolic Remodeling in Unselected ES-2 Cells, Resembling the Metabolic Profile of the Cells of Origin of These cfDNAs

The cfDNA was isolated from the conditioned culture media of ES-2 cells cultured in the absence and presence of glucose during 6 h and 48 h, generating four different variants of cfDNA (Early-cfDNA-NoGluc, Early-cfDNA-Gluc, Late-cfDNA-NoGluc, and Late-cfDNA-Gluc). Subsequently, new ES-2 cells were cultured in the presence of glucose for 48 h and exposed to the four variants of cfDNA, and the conditioned media were analyzed by NMR spectroscopy for exometabolome definition. The PCA score plot showed that different cfDNA variants exerted different effects on ES-2 exometabolome; there is a clear separation of the exometabolome of cells treated with Late-cfDNA-Gluc that cluster together, as opposed to the cells treated with Late-cfDNA-NoGluc that cluster together (Figure 1C). Cells exposed to Late-cfDNA-NoGluc showed accumulation of several metabolites on the supernatant when compared to the data regarding cells treated with Late-cfDNA-Gluc, as depicted in the heatmap (Figure 1D). The treatment with Late-cfDNA-NoGluc decreased the consumption of glucose significantly compared to the control condition *(p* = 0.0089) (Figure 1E). Increased levels of lactate were verified upon exposure to Late-cfDNA-NoGluc when compared to every other experimental condition (Figure 1E). The levels of arginine *(p* = 0.0264)*,* isoleucine *(p* = 0.0299), lysine *(p* = 0.0003), and valine *(p* = 0.0192) in the supernatants of cells treated with Late-cfDNA-NoGluc were significantly increased compared to cells treated with Late-cfDNA-Gluc (Figure 1F). The treatment with Late-cfDNA-Gluc tended to decrease the levels of glycine and threonine compared to the No-DNA condition (Figure 1F). The patterns of production and consumption of the metabolites present in culture media observed upon both Late-cfDNAs-NoGluc and Late-cfDNAs-Gluc exposure resemble the exometabolome of the cells of origin of these cfDNAs. This is highlighted by the heatmap regarding the exometabolome of cells treated with the late variants of cfDNA and the exometabolome of cells of origin, where once can observe the accumulation of amino acids in the supernatants of cells in the absence of glucose or treated with the cfDNA variant that originated from cells in glucose scarcity (Figure 1G).

### 3.3. The Metabolic Remodeling Promoted by Late-cfDNA-Gluc Sustains Cell Proliferation of Unselected Cells, but upon Long-Term Selection, Late-cfDNA-Gluc Induced Quiescence

**Figure 2:**
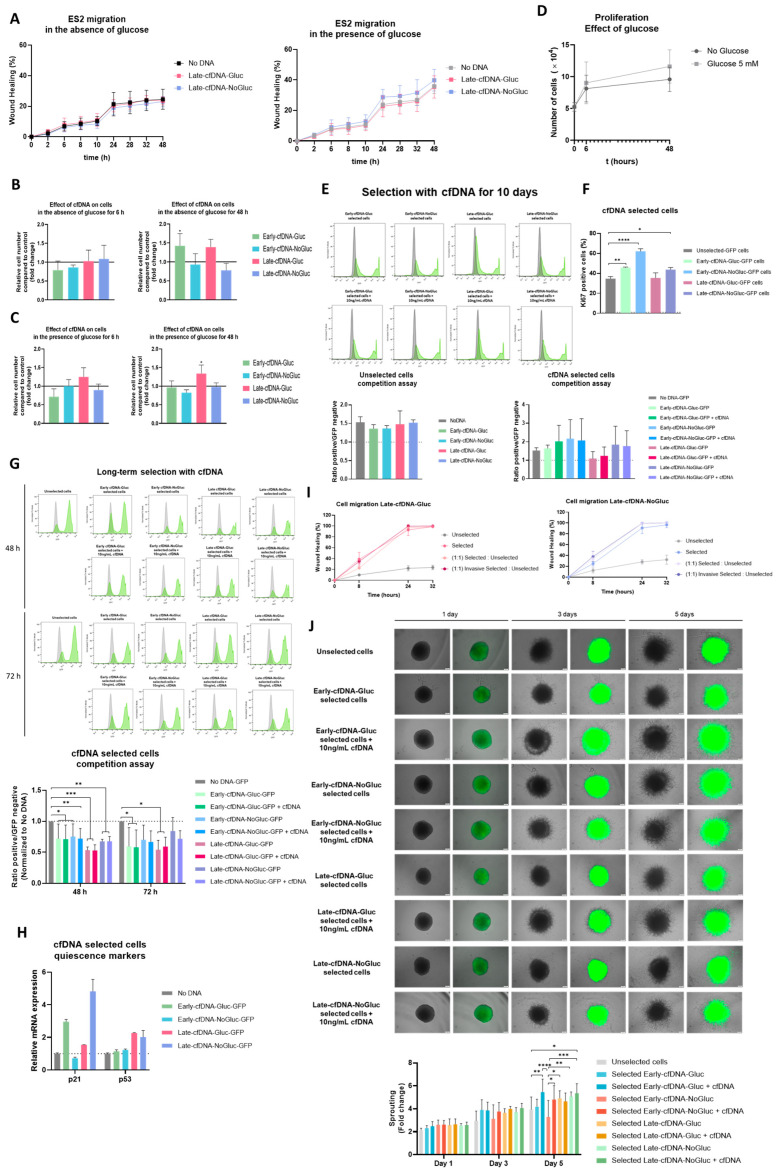
The cfDNA variants have the ability to promote cell proliferation and induce quiescence. Cells were cultured in the presence or absence of glucose and exposed to each variant of cfDNA. (**A**) Quantification of wound closure in control and experimental conditions in the absence and presence of glucose. (**B**) Relative number of cells in the absence of glucose exposed to each variant of cfDNA. (**C**) Relative number of cells in the presence of glucose exposed to each variant of cfDNA for 48 h. (**D**) Proliferation curves of cells in the presence or absence of glucose for 0, 6, and 48 h. (**E**) Competition assays of unselected cells upon cfDNA variants’ stimulation (bottom right) and in selected cells for 10 days with and without cfDNA variants’ exposure (bottom left). (**F**) The positive rate of Ki67 expression in unselected and cfDNA variants’ selected cells analyzed by flow cytometry. (**G**) Competition assays of selected cells for 4 weeks with and without cfDNA variants’ exposure for 48 and 72 h. Results shown as the ratio between GFP-positive (green) and -negative (grey), normalized to the No-DNA condition. (**H**) Relative gene expression of quiescence markers in selected cells for 4 weeks. (**I**) Quantification of wound closure upon selection with Late-cfDNA-Gluc and Late-cfDNA-NoGluc variants. Unselected: Cells that were exposed to cfDNA variants for 32 h with no previous exposure. Selected: Cells that were exposed to cfDNA variants for 32 h with previous exposure to the cfDNA variant for 4 weeks. (1:1) Selected/unselected: Co-culture of selected and unselected cells at a ratio of 1:1 and further exposed to cfDNA variant for 32 h. (1:1) Invasive selected/unselected: Co-culture of unselected and selected invasive isolated cells at a ratio of 1:1 and further exposed to cfDNA variant for 32 h. (**J**) Spheroids sprouting assays of selected cells with and without cfDNA variants’ exposure at 1, 3, and 5 days. Sprouting results are shown as fold change between sprouting area and spheroid core area. Results are shown as mean ± SD. * *p* < 0.05, ** *p* < 0.001, *** *p* < 0.001, and **** *p* < 0.0001. One-way ANOVA was used, followed by Tukey’s test.

The effect of cfDNA on cancer cell proliferation and migration was evaluated in ES-2 cells cultured with no cfDNA (control conditions) and with the four variants of cfDNA, also in the absence or presence of glucose, during 6 h and 48 h (Figure 2). Regarding cell migration, in the absence or presence of glucose, no significant differences were found upon exposure to cfDNA variants (Figure 2A).

For cell proliferation, cells were evaluated in the absence of glucose upon exposure to the cfDNA variants (Figure 2B). In the absence of glucose for 6 h, the exposure to Early-cfDNA-Gluc induced a trend toward decreasing cell proliferation compared with the control; this trend was rescued upon 48 h of exposure, and a significant increase in cell proliferation was observed (Figure 2B). The stimulus with Late-cfDNA-Gluc induced a trend toward increasing the proliferative capacity of ES-2 cells after 48 h. Cell proliferation was not altered due to the exposure to the Early- and Late-cfDNA-NoGluc variants. In the presence of glucose, the exposure to Late-cfDNA-Gluc induces a trend toward increasing cell proliferation after 6 h compared with the control group, and this tend became significant at 48 h (Figure 2C). Cell proliferation was not altered due to the exposure to the other cfDNA variants. In order to better understand the different stimuli upon proliferation induced by the different cfDNA variants and their association with the glucose deprivation, we assessed the impact of glucose bioavailability on the proliferation capacity of ES-2 cells. We observed that glucose scarcity induced a slower proliferative rate in ES-2 cells through the 48 h of incubation (Figure 2D). Interestingly, the proliferation rate was higher at incubations of 6 h compared to the longer incubation time period (no glucose: slope for 6 h = 2.881; slope for 48 h = 1.4524; and Glucose: slope for 6 h = 3.781; slope for 48 h = 2.5857). In order to clarify the role of cfDNA variants in controlling proliferation, unselected ES-2 was co-cultured together with GFP-ES-2 previously selected with each one of the cfDNA variants. It was observed that, upon 10 days of exposure to the cfDNA variants, Late-cfDNA-Gluc decreased the ratio of selected vs. unselected cells (Figure 2E), indicating that unselected cells upon Late-cfDNA-Gluc stimulus proliferate more, and the selected become more quiescent, as confirmed by the lower levels of Ki67 marker in Late-cfDNA-Gluc-selected cells compared to the other cfDNA variants (Figure 2F). Selected cells upon Early-cfDNA-NoGluc stimulus showed the highest levels of Ki67 marker (Figure 2F). Interestingly, long-term and continuous exposure to all cfDNA variants significantly decreased the ratio of selected cells when compared to the No-DNA-GFP condition (Figure 2G). We measured the mRNA levels of *CDKN1A* and *TP53* genes since they are key regulators of cell cycle arrest and quiescence [44,45]. The possible induced quiescent state was confirmed by the increased relative mRNA levels of *CDKN1A* and *TP53* genes shown by the selected cells, encoding p21 and p53 proteins, respectively (Figure 2H). Since cancer cells’ quiescence favors chemoresistance [47], the next step was to explore if cfDNA variants could select more resistant cells. In order to assess the potential role of cfDNA on the regulation of the migration capacity, unselected ES-2 cells were co-cultured together with GFP-ES-2 cells previously selected with the Late-cfDNA variants that were further selected to isolate the more invasive cells. The results showed that selection with both Late-cfDNA variants induced an increase in the migratory capacity of ES-2 cells (Figure 2I). Spheroid invasion assays allow for the assessment of the invasive behavior of the cells by embedding 3D cancer cell cultures in a matrix [48,49]. Therefore, we further evaluated the pro-migratory and invasive potential of the cfDNA variants by analyzing the sprouting of spheroids of co-cultures of unselected ES-2 cells and previously selected GFP-ES-2 cells. Our results showed that, on day 3, the presence of previously selected cells induced a trend toward increasing the sprouting capacity of the spheroids, except for the Early-cfDNA-NoGluc (Figure 2J). Interestingly, on day 5, the selection of GFP-ES-2 cells with Early-cfDNA-Gluc variant abrogated this trend; however, the presence of the stimulus of Early-cfDNA variants further increased the sprouting capacity of the spheroids compared to the unselected spheroids. No preferential localization of selected GFP-ES-2 cells on the leading edge was detected (Figure 2J).

### 3.4. All cfDNA Variants Promoted Cisplatin Resistance in ES-2 Cells, and Cysteine Protects Cells from Cisplatin in Hypoxia-Mimicked Conditions, Potentiating the Early-cfDNA-Gluc Protection

In solid tumors, cancer cells are exposed to regions of hypoxia, and it was previously demonstrated by our team that cysteine supplementation conferred a protective effect in ovarian cancer cells from hypoxia-induced death, and that cysteine is critical for chemoresistance [33,34,50]. Herein, we address whether cfDNA also plays a protective role in tumor cells, disclosing the effect of each cfDNA variants on cell death upon cisplatin exposure in both normoxia and hypoxia (CoCl_2_) conditions upon cysteine supplementation.

**Figure 3:**
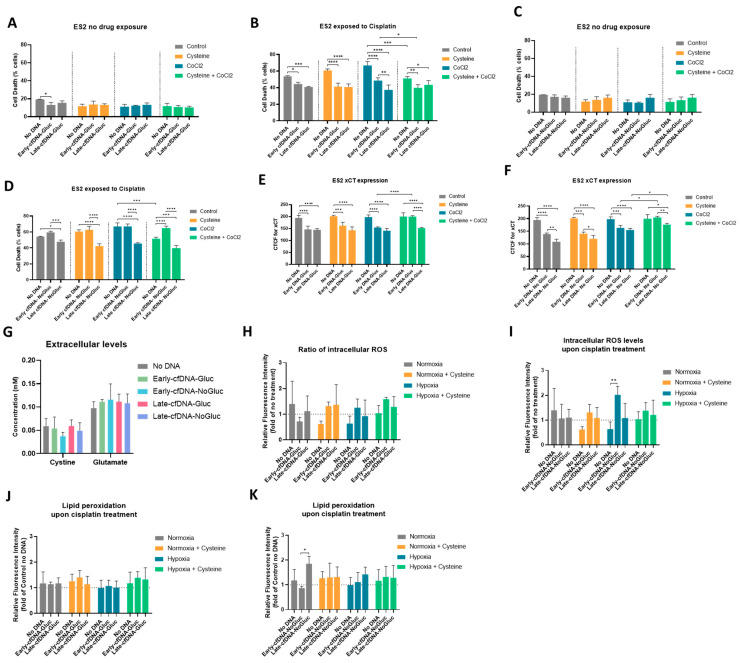
CfDNA variants have the ability to induce cisplatin resistance. (**A**–**D**) Percentage of cell death analyzed by flow cytometry using Annexin V and PI of cells. (**A**,**C**) Cell death evaluated in a drug-free context for Early-cfDNA-Gluc and Late-cfDNA-Gluc variants (**A**) and Early-cfDNA-NoGluc and Late-cfDNA-NoGluc variants (**C**). (**B**,**D**) Cell death evaluated upon cisplatin exposure for Early-cfDNA-Gluc and Late-cfDNA-Gluc variants (**B**) and Early-cfDNA-NoGluc and Late-cfDNA-NoGluc variants (**D**). xCT expression by immunofluorescence for Early-cfDNA-Gluc and Late-cfDNA-Gluc variants (**E**) and Early-cfDNA-NoGluc and Late-cfDNA-NoGluc variants (**F**). (**G**) Extracellular levels of cystine and glutamate. (**H**,**I**) Levels of intracellular ROS for Early-cfDNA-Gluc and Late-cfDNA-Gluc variants (**H**) and Early-cfDNA-NoGluc and Late-cfDNA-NoGluc variants (**I**). (**J**,**K**) Levels of lipid peroxides for Early-cfDNA-Gluc and Late-cfDNA-Gluc variants (**J**) and Early-cfDNA-NoGluc and Late-cfDNA-NoGluc variants (**K**). Results are shown as mean ± SD. * *p* < 0.05, ** *p* < 0.001, *** *p* < 0.001, and **** *p* < 0.0001. One-way ANOVA was used, followed by Tukey’s test.

Assessing the effect of cfDNA isolated from cells cultured with glucose (Early- and Late-cfDNA-Gluc) in the absence of cisplatin, ES-2 cells showed decreased cell death when treated with Early-cfDNA-Gluc in control conditions (normoxia) (Figure 3A). The hypoxia-mimicked conditions and presence of cysteine in normoxia or in hypoxia did not affect cell death levels in a drug-free context (Figure 3A). In cisplatin exposure, considering all conditions of normoxia, hypoxia, and cysteine, both Early- and Late-cfDNA-Gluc variants promoted decreased cell death when compared with the respective No-DNA control (Figure 3B). No protection effect from cysteine exposure was observed in normoxia (Figure 3B). However, considering the No-DNA control conditions in the absence of cfDNA and in hypoxia, cell death increased upon cisplatin, but cysteine rescued this effect and decreased cell death (*p* = 0.0001) (Figure 3B). The same protective effect was observed in cells exposed to Early-cfDNA-Gluc (*p* = 0.0324) (Figure 3B).

Regarding the effect of cfDNA isolated from cells cultured without glucose (Early- and Late-cfDNA-NoGluc), in the absence of cisplatin, no differences were found concerning normoxia, hypoxia, or cysteine presence (Figure 3C). In the presence of cisplatin, a protective effect of Late-cfDNA-NoGluc was observed, highlighted by the decreased cell death levels compared with the No-DNA conditions (Figure 3D). Regarding the presence of cysteine, a trend for a concomitant protective effect was observed between cysteine and Late-cfDNA-NoGluc exposure in both normoxia and hypoxia (Figure 3D).

### 3.5. xCT Cyst(e)ine Antiporter May Be Involved in the Concomitant Protective Effect Between Cysteine and cfDNA

The results showed that cysteine protected ES-2 cells from cisplatin in hypoxia, potentiating the Early-cfDNA-Gluc protection. Furthermore, a correlation between the expression of cyst(e)ine antiporter xCT and chemoresistance was described [38], and we further evaluated whether the mechanism through which DNA was promoting cisplatin resistance was associated with the expression of xCT. We assessed the xCT protein levels on ES-2 cells upon exposure to each cfDNA by immunofluorescence.

For No-DNA conditions, no differences were observed in xCT expression levels, independently of normoxia, hypoxia, and cysteine presence (Figure 3E,F). The results showed that all cfDNA variants led to a significant decrease in xCT protein levels compared with No-DNA conditions (Figure 3E,F). However, in hypoxia-mimicked conditions cysteine promoted the rescue of xCT expression in cells exposed to Early-cfDNA-Gluc (*p* < 0.0001), Early-cfDNA-NoGluc *(p* < 0.0001), and Late-cfDNA-NoGluc (*p* = 0.0353) (Figure 3E,F).

The antiporter system xCT imports cystine, the oxidized form of cysteine, into cells, while it counter-transports glutamate [38]; hence, we assessed the modulation of these amino acids upon stimulation with the four different variants of cfDNA. No significant differences were detected; however, the variant Early-cfDNA-NoGluc induced a trend of decreasing cystine levels on the supernatants (Figure 3G).

The mechanism of action of cisplatin is based on the formation of DNA adducts inhibiting cell growth, along with the generation of intracellular reactive oxygen species (ROS), ultimately inducing cell death [51]. Herein, we addressed whether the protective role of the cfDNA variants upon cisplatin exposure was associated with a decrease in the levels of intracellular ROS. The results showed that, upon cisplatin treatment, in normoxia conditions, cfDNA variants’ exposure induced a trend toward decreasing the intracellular levels of ROS compared to the No-DNA condition (Figure 3H,I). Cysteine supplementation induced a protective role by reducing the levels of intracellular ROS only in the No-DNA condition. Regarding hypoxic conditions, the data showed that, in the absence of DNA stimulation, cisplatin treatment tended to decrease ROS levels, which were reverted upon cfDNA variants’ exposure, showing a significant increase in cells exposed to Early-cfDNA-NoGluc *(p* = 0.0098) (Figure 3I). Interestingly, in hypoxia-mimicked conditions, cysteine supplementation resulted in a trend toward increasing the levels of intracellular ROS, except for cells stimulated with Early-cfDNA-NoGluc, where an opposite trend was observed (Figure 3H,I). The increased levels of ROS induced by cisplatin react with membrane lipids, increasing lipid peroxidation, which can disrupt membrane structure and function, potentially leading to cell death, so we further evaluated the levels of lipid peroxidation [52,53]. For No-DNA conditions, no differences were observed in lipid peroxidation levels, independent of the presence of normoxia, hypoxia, and cysteine (Figure 3J,K). Under normoxia, cells stimulated with Late-cfDNA-NoGluc variant showed significantly increased levels of lipids peroxides compared to the variant Early-cfDNA-NoGluc, which was the only variant that showed a trend of decreasing lipid peroxidation levels upon cisplatin treatment (Figure 3K). Cysteine supplementation rescued the effect induced by the Late-cfDNA-NoGluc variant (Figure 3D).

### 3.6. All cfDNA Variants Decreased Toll-like Receptor 9 (TLR9) Levels

**Figure 4:**
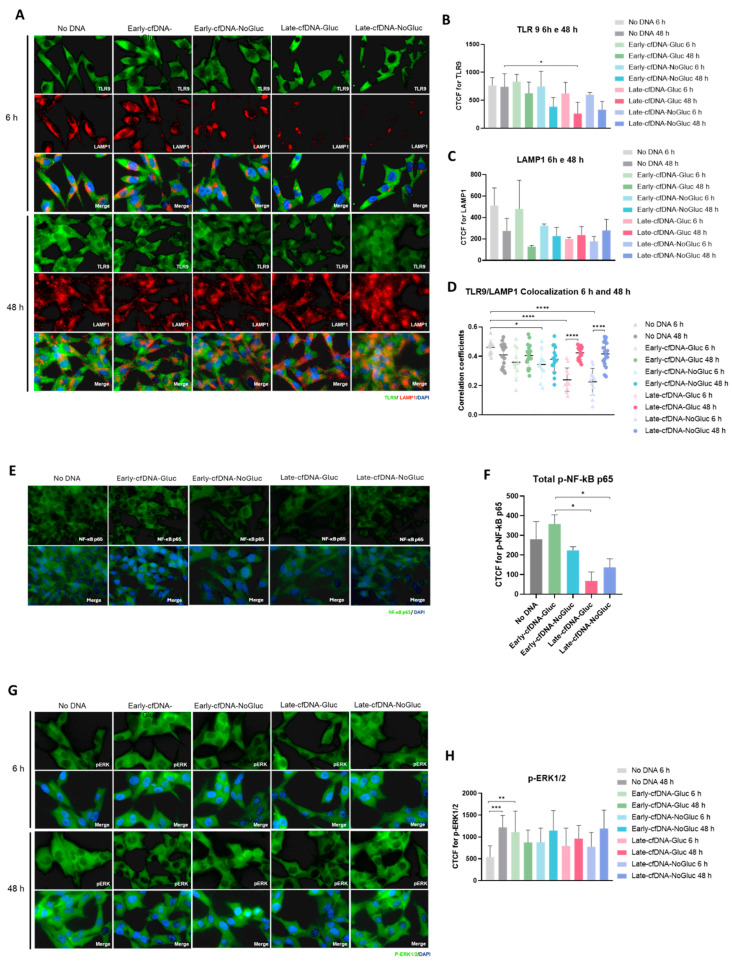
CfDNA variants decrease TLR9 protein levels. Cells were cultured in the presence of glucose and exposed to each variant of cfDNA. (**A**) Immunofluorescence for detection of TLR9 (green) and LAMP1 (red), which were quantified using ImageJ program (**B**,**C**), respectively. (**D**) Pearson’s correlation coefficient for the colocalization analysis between TLR9 and LAMP1 proteins. (**E**) p-NF-кB expression by immunofluorescence. (**F**) Total p-NF-кB immunofluorescence quantification (CTCF) using the ImageJ program. (**G**) p-ERK1/2 expression by immunofluorescence. (**H**) Total p-ERK1/2 immunofluorescence quantification (CTCF) using the ImageJ program. Results are shown as mean ± SD. * *p* < 0.05, ** *p* < 0.001, *** *p* < 0.001, and **** *p* < 0.0001. One-way ANOVA was used, followed by Tukey’s test.

TLR9 is a DNA receptor that can recognize mammalian DNA containing unmethylated cytosine–guanosine (CpG) dinucleotides [54] and plays a key role in mediating DNA effects on cancer cells. TLR9 is primarily located in intracellular vesicles within the endoplasmic reticulum and Golgi apparatus that, upon stimulation by ligands, translocate to endolysosomes [55]. We assessed the expression of TLR9 protein and lysosome-associated membrane protein 1 (LAMP1) and explored the grade of colocalization among these proteins upon stimulation with each cfDNA variant for 6 and 48 h. Regarding the colocalization of TLR9 and LAMP1 proteins, a significant decrease was observed upon 6 h of exposure to all variants except for Early-cfDNA-Gluc (Figure 4A,D). Interestingly, the exposure to the Late-cfDNA variants promoted a significant increase in TLR9 and LAMP1 colocalization from 6 h to 48 h (Figure 4A,D). The stimulation of ES-2 cells with the cfDNA variants induced a trend toward decreased TLR9 protein levels from 6 h to 48 h (Figure 4B). A significant decrease in TLR9 protein levels was detected upon 48 h of exposure to the Late-cfDNA-Gluc compared to the No-DNA condition (Figure 4B). Exposure of ES-2 cells to Late-cfDNA variants induced a trend toward decreased levels of LAMP1 protein from 6 h to 48 h, opposing the trend induced by the stimulus with the Early-cfDNA variants and No-DNA condition (Figure 4C). Interestingly, the exposure to cfDNA variants induced a trafficking of LAMP1 protein, which, at 6 h, was mainly accumulated in the perinuclear area of ES-2 cells and, at 48 h, was found dispersed in the cytoplasm of the cells (Figure 4A). TLR9 activation is strongly associated with the production and release of cytokines, where the stimulation of TLR9 triggers its interaction with Myeloid differentiation factor 88 (MyD88), activating signaling cascades that lead to the activation of the nuclear factor-kB (NF-кB) or extracellular signal-regulated kinase 1/2 (ERK1/2), which controls the expression of inflammatory cytokines [39,40]. We further assessed the levels of p-NF-κB p65 upon exposure to each cfDNA variant for 48 h. ES-2 cells showed increased levels of p-NF-κB p65 upon exposure to the Early-cfDNA-Gluc variant compared to both Late-cfDNA-NoGluc and Late-cfDNA-Gluc variants (Figure 4E,F). Additionally, we evaluated the levels of p-ERK1/2 upon stimulation with each cfDNA variant for 6 and 48 h. After 6 h, the exposure to Early-cfDNA-Gluc variant induced a significant increase in p-ERK1/2 levels compared to the No-DNA condition, and the remaining cfDNA variants promoted a trend toward increasing its levels (Figure 4G,H). After 48 h, all conditions induced a trend toward increased p-ERK1/2 protein levels, except for the Early-cfDNA-variant, which induced a trend toward a decrease in its levels (Figure 4G,H).

## 4. Discussion

Clinical relevance of cfDNA in oncology has received considerable interest over the past few years. Different studies have demonstrated the potential use of cfDNA as a biomarker for cancer follow-up and management, including diagnosis, prognosis, and monitoring [13,14,15]. However, the biological significance of cfDNA in cancer establishment and progression has not yet been elucidated. The regulation of cancer cell functioning as a systemic process [56] requires the activity of several regulators, and cfDNA found in the peripheral blood of cancer patients has been demonstrated to activate signaling pathways [57], namely through toll-like receptors (TLRs) [58].

Conceptually, cfDNA may have a role in the adaptive capacity of cancer cells to stressful conditions. These hurdles are presented during the course of the disease and promote selection of cancer cells that are competent to carry on progression [59]. Tumor heterogeneity limits the adaptation to the tumor microenvironment, and sensitivity and resistance are exhibited differently amongst cancer cell subsets within a tumor [60]. Accordingly, cfDNA can serve as a means of communication between cancer cells to coordinate adaptation. In this study, the role of cfDNA derived from ES-2 cells, an OCCC cell line, on the metabolic remodeling and cisplatin resistance was explored, considering different glucose bioavailability and duration of culture. Therefore, we considered no glucose culture to be an unfavorable, stressful condition, and glucose culture an ideal, favorable condition, and cfDNA release can represent a consequence of suffering or an attempt to survive. ES-2 cells were exposed to these culture conditions for 6 h and 48 h in order to promote cell adaptation.

Because glucose is considered an essential fuel for tumor progression, we evaluated the differences in the metabolic reprogramming induced by the glucose bioavailability. ES-2 cells showed similar metabolic profiles at 6 h of being in the presence or absence of glucose; however, after 48 h, differences in glucose bioavailability induced distinct metabolic profiles on ES-2 cells (Figure 1A), showing increased amino acids consumption and the production of lactate and citrate when glucose is present in the media (Figure 1B). The synthesis of lactate associated with the consumption of glucose indicates glucose catabolism, suggesting that the glycolysis pathway was activated.

Cancer cells produce fatty acids not only to be able to divide and restore the plasma membrane but also to guarantee the regulation of the function of several transporters and receptors [61,62]. In order to fulfill this lipid requirement, the cells increase citrate synthesis, and the tricarboxylic acid (TCA) cycle will most likely need to be reversed and truncated [63]. It can be extrapolated that to sustain the synthesis of high levels of citrate, cancer cells exchange their metabolic routes to glycolysis [63]. It was disclosed that citrate can be supplied to cancer cells by the tumor microenvironment, where neighboring cells synthesize and release citrate to the extracellular media, increasing epithelial-to-mesenchymal transition and, thus, promoting metastatic progression [64]. It was also described that the presence of extracellular citrate is associated with a reduction of intracellular amino acid levels, indicating a catabolic switch of cancer metabolism [64]. Accordingly, our results showed that the presence of glucose promoted the synthesis and release of citrate to the extracellular media, and concomitantly lower levels of extracellular amino acids were detected (Figure 1B), suggesting its increased consumption, proposing its catabolism. Amino acids play a role in biosynthesis, acting also as energy sources and helping in the maintenance of redox balance. Glutamine was found to be absent after 48 h, indicating its uptake by cells whether in the presence or absence of glucose. Glutamine is considered a crucial amino acid for tumor cells, as it is suggested to be the most rapidly consumed amino acid in tumor cells [65]. It is an important amino acid for fueling the TCA cycle to sustain ATP production, the maintenance of redox homeostasis, and signal transduction processes [66]. Glutamine is converted to glutamate, which can be further converted to α-ketoglutarate, also referred to as 2-oxoglutarate, fueling the TCA cycle; cells in the presence of glucose showed increased levels of α-ketoglutarate (Figure 1B).

Additionally other amino acids can act as alternative fuel sources for cells to optimize nutrient use during the progression of the tumor [67]. Branched-chained amino acids (BCAAs), which include valine, leucine, and isoleucine, showed to be more taken up by the cells that were in the presence of glucose. They, besides acting as alternative sources of organic molecules that can fuel the TCA cycle [68], can also function as nitrogen donors, which are important in the synthesis of nucleotides [69,70], playing a crucial role in cancer cell growth. Arginine extracellular levels were also found decreased when cells were in the presence of glucose for 48 h. L-arginine is a precursor of polyamines, which are critical for tumor progression by increasing DNA synthesis and facilitating cell proliferation, and they confer protection to nucleic acids from damage, enabling DNA stability [71,72,73]. The amino acid L-arginine is also the precursor of nitric oxide (NO), which is a signaling molecule that regulates several cellular processes, tumor growth, promotion of extracellular matrix remodeling, and angiogenesis. It was described that the pathway L-arginine/NO plays a major role in ovarian cancer progression [74,75].

Moreover, under the same condition of glucose availability, the cfDNA variants exerted different effects on ES-2 cells exometabolome (Figure 1C). Opposite effects were induced by the exposure to the variants Late-cfDNA-Gluc and Late-cfDNA-NoGluc on the cells’ exometabolome (Figure 1C,D), proposing that, in a tumor context, cells under different environments with distinct metabolites bioavailability may release different signals to the neighboring cells that are capable of modulating their metabolic profiles. Interestingly, the exometabolome upon the stimulation with the Late-cfDNA-NoGluc variant showed significantly decreased consumption of glucose compared to No-DNA cells (Figure 1E), supporting the possibility of cells, upon a nutritional deprivation, releasing cfDNA, which provides a stimulus for neighboring cells to spare nutrients, even when they are cultured with glucose. Late-cfDNA-NoGluc, besides decreasing the consumption of glucose, also promoted the accumulation of extracellular lactate (Figure 1E). Growing evidence shows that lactic acidosis, which results from the intensive secretions of lactate and protons, plays a key role in tumor physiology promoting cancer cell resistance to glucose deprivation [76]. Additionally, acidosis promotes tumor cell survival under glucose deficiency by causing cancer cells to enter a metabolically dormant but energetically economic state, limiting glucose and ATP consumption and reprogramming tumor cell metabolism [77]. This agrees with the induction of quiescence in ES-2 cells selected under Late-cfDNA-NoGluc exposure, since quiescent cells present low metabolic levels [78]. Studies have suggested that acidosis-induced metabolic reprogramming involves two mechanisms of action: inhibition of glycolysis and of rRNA/protein synthesis, thus preventing the ATP consumption [77,79,80].

Interestingly, the stimulation of ES-2 cells with the variants Late-cfDNA-Gluc and Late-cfDNA-NoGluc induced different patterns of consumption of amino acids that resemble the exometabolome of the cells of origin of these cfDNAs (Figure 1D,G). Significantly increased extracellular levels of arginine, leucine, lysine, and valine were detected upon treatment with Late-cfDNA-NoGluc compared to the exposure to the variant Late-cfDNA-Gluc. Leucine and valine, were both diminished in Late-cfDNA-Gluc and increased in Late-cfDNA-NoGluc (Figure 1F), showing an interconnected dynamic, as described for BCAA [81].

Our results propose that cfDNA transfers information inherent to the cells of origin; therefore, the phenotypes of new cells exposed to the cfDNA variants are altered according to this diverse information, independently of the fact that new cells were cultured in glucose-supplemented media upon the exposure cfDNA variants. The amino acids profile proves this dependence on the profile of the cells of origin of cfDNA variants. The heatmap concerning the exometabolome of cells exposed to Late-cfDNA-Gluc and Late-cfDNA-NoGluc variants and the respective conditioned media of origin (Figure 1G), demonstrated that glucose is imported, glycolysis is activated, and lactate is produced. Furthermore, all cells showed increased levels of glutamate, pyroglutamate, and citrate upon glucose availability, as well as pyruvate and alanine. However, the amino acid consumption showed to be more variable and dependent on the cfDNA stimulus, resembling the origin of the cfDNA variants (Figure 1G).

Regarding the phenotypical regulation, it was observed that the cfDNA variants impacted cancer cell features differently. Cell proliferation is intimately associated with the promotion of tumor heterogeneity [82], because better-adapted cells proliferate at a higher rate and gain an advantage over maladapted cells. It was observed that Early-cfDNA-NoGluc and Late-cfDNA-NoGluc did not induce any alteration in the proliferation rate of ES-2 cells, independent of the availability of glucose and the culture duration. This suggests that cfDNA released under the imposed stressful conditions was not efficient in regulating proliferation. Contrarily, after 48 h of exposure, Early- and Late-cfDNA-Gluc variants significantly stimulated cell proliferation in the absence and presence of glucose, respectively (Figure 2B,C). In ideal culture conditions with glucose, a cell culture starts to actively proliferate, and it makes sense that cells coordinate and cross-stimulate the cell culture’s proliferation. Thus, the released Early-cfDNA-Gluc showed to have a pro-proliferative effect, making it able to stimulate proliferation in cells cultured in the disadvantageous no-glucose condition (Figure 2B). This process involves adaptation; consequently, the differences are significant after 48 h of culture. Late-cfDNA-Gluc also showed a pro-proliferative effect, highlighted by a trend toward increasing proliferation in the absence of glucose at 48 h and in the presence of glucose at 6 h, in addition to the significant effect in the presence of glucose at 48 h (Figure 2B,C). A growth curve indicates the growth characteristics of a cell line under different conditions. Our results indicated that the availability of glucose increased the proliferative capacity of ES-2 cells, and the values of the slopes of the growth curves indicated that, at 6 h, cells were proliferating at an increased rate compared to 48 h, since the slope value is proportional to the division rate; therefore, when the slope of the curve reaches is maximum value, cells are in the exponential growth phase and dividing at their highest rate [83]. Thus, the different extent to the pro-proliferative effects concerning the variants Early- and Late-cfDNA-Gluc may be a result of the different proliferative phases from which they were isolated. The Late-cfDNA-Gluc was isolated after 48 h from a cell culture that was already proliferating at a lower rate when compared to the timepoint of 6 h, at which Early-cfDNA-Gluc was isolated (Figure 2D). This fact can justify the lower efficacy of Late-cfDNA-Gluc to significantly stimulate ES-2 cell proliferation in the absence of glucose, as compared to Early-cfDNA-Gluc. Despite this, Late-cfDNA-Gluc efficiently sustains a significant proliferation rate of ES-2 cell cultured during 48 h with glucose. In order to support cell proliferation, cells must rewire their metabolism so that they are able to support their bioenergetic and biomass requirements [84]. Accordingly, the capacity of cfDNA in regulating and promoting cell proliferation, as well as the metabolic remodeling, seems to be strongly dependent on the conditions of the microenvironment surrounding the cells that are releasing it. Considering the stimulation of both Late-cfDNA variants in cells that are in the presence of glucose, our findings showed that the Late-cfDNA-Gluc variant, in contrast to the variant Late-cfDNA-NoGluc, sustained the metabolic demands of its pro-proliferative effect on ES-2 cells by promoting a metabolic rewiring that induced a trend toward increased glucose uptake and the increased consumption of several amino acids that are important to the synthesis of macromolecules required for DNA replication, cell division, and ultimately supporting tumor growth (Figure 2B,C).

Regarding cell migration and the influence of Late-cfDNA-NoGluc and Late-cfDNA-Gluc, no alteration was observed in the wound-healing assay performed in the absence of glucose (Figure 2A), but a trend toward increasing migration was observed in cells cultured with glucose and exposed to Late-cfDNA-NoGluc (Figure 2A). cfDNA isolated from cells cultured in stressful conditions seems to stimulate ES-2 cell migration. This is in agreement with studies showing that cells exposed to nutrient scarcity may increase migration and activate micropinocytosis, as reviewed [85,86]. Therefore, cfDNA isolated from cells under starvation-forced migration may promote a migratory remodeling in other cancer cells. The PI3K/AKT pathway is crucial in the control of cell migration and invasion [87], and it also seems to be relevant in this starvation-related migration [85]. This can be another indication that cfDNA-dependent signaling through TLR9 may be involved in this process, since the PI3K pathway is targeted by TLR9 [88,89]. Moreover, glucose starvation induces cancer cell migration in processes involving the upregulation of MMP9, whose activation depends on NRF2 [90]. Interestingly, NRF2 is the master controller of oxidative stress, activating the expression of proteins accounting for cytoprotection and metabolic remodeling to cope with redox imbalance [91]. Furthermore, cancer cells cultured in the absence of present oxidative stress, because glucose is essential for redox control, since it sustains the pentose phosphate pathway (PPP), which is crucial in redox glutathione regeneration [92]. However, quiescence was induced by a long-term exposure to Late-cfDNA-Gluc variant (Figure 2E,F). In the bulk of ES-2 cells, the immediate effect of Late-cfDNA-Gluc was the stimulation of proliferation, but some subsets of ES-2 cells undergo a quiescent process. Increasing the time of continuous exposure led to the decreased ratio of selected cells compared to the No-DNA-GFP condition, inferring a decreased proliferative rate of selected cells, suggesting the induction of a quiescent state by all cfDNA variants (Figure 2G). An inhibitory effect on cell cycle and division induced by DNA was already reported on yeasts [93,94]; however, in a cancer-cell context, there are no reports regarding a pro-quiescent role of cfDNA. Furthermore, the interplay between cell proliferation rate and sugar metabolism has been reported as a crucial factor explaining the phenomenon known as SICD (sugar-induced cell death) in yeast [37]. This is a key subject that warrants additional inquiry in the cancer context. The senescent growth arrest is achieved through induction of tumor-suppressor p53 (*TP53*), which regulates the expression of cyclin-dependent kinase inhibitors such as p21, which plays a critical role in the G1 exit program; therefore, quiescent cells are associated with increased levels of p53 and p21 [44,45]. Cells selected by the long-term exposure to cfDNA variants showed increased mRNA levels of quiescence markers, p21 and p53 encoding genes, with the exception of Early-cfDNA-NoGluc selected cells (Figure 2H), which, interestingly, were the cfDNA variant that did not induce cisplatin resistance (Figure 3D). Quiescent cells are more protected from anti-cancer therapy, and they are considered to be the pawns of cancer metastasis and disease relapse [47,95]. Few cells in a tumor are in fact quiescent cancer stem cells; however, they are powerful enough to bring back disease in a more aggressive version [45,96,97]. Therefore, increasing the knowledge about entry into and exit from quiescence in cancer must be a goal to avoid disease progression and relapse. Since the tumor microenvironment downregulates and selects only a small subset of cells to become dormant/quiescent cells, the quiescence was only detected in cells selected by cfDNA variants in a long-term fashion, by allowing the increase in these cells subsets and consequently its detection. ES-2 cells, upon long-term selection, showed a more migratory and invasive behavior (Figure 2I,J). The initial step of the formation of metastatic tumors relies on the capacity of cancer cells to invade the surrounding tissue [98]. The metastasis-initiating cells are described as being less proliferative but more invasive, which is in agreement with our results [99]. According to the migration–proliferation dichotomy, when cells proliferate, their migratory ability is temporarily inhibited, only to be reactivated once cell division ceases.

Acquired chemoresistance is a major issue in oncology. The conventional treatment for ovarian cancer is debulking surgery, followed by radiation therapy and/or chemotherapy, with platinum- and taxane-based drugs being more commonly used to treat ovarian cancer. It is clear that ovarian-cancer patients develop acquired resistance to platinum- and taxane-based therapies, but the molecular mechanisms underlying chemoresistance in ovarian cancer are poorly understood [19,25,100]. It was reported by our team the critical role of cysteine in ES-2 cells’ adaptation to hypoxia, which favored chemoresistance [33,34,50]. Our results confirm the protective effect of cysteine under hypoxic conditions upon cisplatin exposure (Figure 3B,D). Cysteine’s role in cancer cells’ survival was already linked to its activity as a precursor of the antioxidant glutathione (GSH), and the elevated GSH levels were connected to chemotherapeutic resistance [101,102,103]. The transcription factor NRF2, which is a critical regulator of the antioxidant response, regulates the expression of enzymes involved in GSH synthesis [104]. Additionally, our data suggest a protective effect of cfDNA under cisplatin treatment. Interestingly, it was reported that, during oxidative stress, oxidized cfDNA can be transferred into the cancer cells, and it has the capability of stimulating antioxidant mechanisms and inducing the transcription factor NRF2 expression [105,106,107]. We found that all cfDNA variants promote cisplatin resistance, except for the Early-cfDNA-NoGluc, which maintained or significantly increased cell death compared to the No-DNA control, further abolishing the protective effect conferred by cysteine in conditions of hypoxia (Figure 3D). Interestingly, this variant induced a trend toward decreasing the extracellular levels of cystine, hinting at a distinct effect on cysteine metabolism compared to the remaining cfDNA variants (Figure 3G). However, the differential modulation of the cysteine metabolism may not be dependent on the regulation of xCT expression since no major differences in its expression were found when compared with the remaining cfDNA variants, and no major differences were found in the extracellular glutamate levels either, which were similar to the other variants (Figure 3E–G). While xCT has gathered much interest in cancer metabolism, cystine can be taken up by the cells through the activity of other transporters, including solute carrier family 3 member 1 (rBAT, *SLC3A1*), which does not require the simultaneous exchange with glutamate [108]. Furthermore, despite the described correlation between the expression of xCT transporter and chemoresistance [38,109], our results indicate that the protective effect of cfDNA does not rely on xCT expression and import of cysteine, since stimulation with all variants of cfDNA decreased xCT protein levels when compared to the No-DNA condition, except in hypoxia-mimicked conditions supplemented with cysteine, where both Early-cfDNA variants rescued xCT expression (Figure 3E,F). Again, evidence supporting the role of cfDNA in chemoresistance is the induction of quiescence by the Late-cfDNA-Gluc variant (Figure 2E,F). Interestingly, the selection with the variant Early-cfDNA-NoGluc induced the highest levels of Ki67 marker (Figure 2F).

TLR9 is primarily found in intracellular vesicles within the endoplasmic reticulum, and upon stimulation by ligands, including CpG dinucleotides, it translocates to late endolysosomes via the Golgi complex residing in LAMP-1 positive vesicles [55,110]. Our results showed that Early-cfDNA-Gluc decreased the levels of LAMP1-late-endolysosomes after 48 h of exposure (Figure 4A,C), in which TLR9 is placed upon activation [110]. Late-cfDNA-NoGluc induced a trend, and Late-cfDNA-Gluc significantly decreased the levels of TLR9 and kept the levels of LAMP1 (Figure 4B,C), indicating the maintenance of late endolysosomes. Moreover, the decreased levels of TLR9 due to Late-cfDNA-Gluc exposure compared to No-DNA condition can be due to proteolysis, a process that is needed for the activation of TLR9 cascade [111,112]. Thus, the decreased TLR9 levels upon 48 h of stimulation with all cfDNA variants compared to No-DNA condition but also regarding the protein levels detected at 6 h (Figure 4B) may be a result of decreased recognition of TLR9 protein by the antibody due to proteolysis. However, the co-localization index between TLR9 and LAMP1-late-endosomes increased in cells exposed to Late-cfDNA-Gluc and Late-cfDNA-NoGluc from 6 to 48 h (Figure 4A,D), suggesting that TLR9 is continuously placed in LAMP1-late-endolysosomes. Unmethylated CpG oligodeoxynucleotides are recognized by TLR9, which induces the activation of signaling cascades including ERK1/2 MAPKs [40,113,114]. The significant increase in p-ERK1/2 levels upon Early-cfDNA-Gluc variant stimulus and the trend toward increasing upon exposure to the remaining variants (Figure 4H) suggest an early response upon recognition of cfDNA and the consequent activation of TLR9. Regarding the Late-cfDNA-Gluc effect on ES-2 cells, TLR9 can underlie the cancer-favoring phenotypical changes observed, such as the metabolic remodeling and the pro-proliferation stimulus in unselected cells and the more prone quiescence induction in ES-2 cells long-term selected by this cfDNA variant, which ultimately supports chemoresistance in a cysteine-dependent manner.

**Figure 5:**
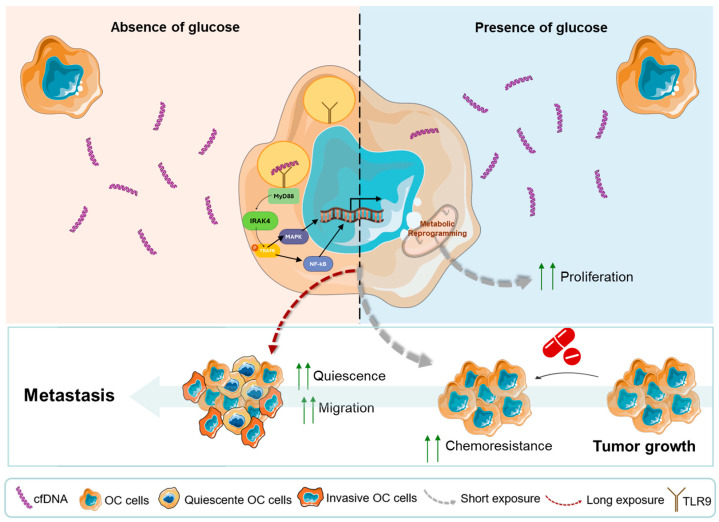
The cfDNA biological role in tumor growth and systemic cancer spread. The short-term exposure of ovarian cancer (OC) cells to cfDNA derived from more favorable conditions promotes a metabolic remodeling supporting cell proliferation, accounting for tumor growth. The cfDNA confers a protective effect against chemotherapeutic agents, driving chemoresistance. Long-term stimulation with cfDNA drives a subset of OC cells to enter a quiescent state which is simultaneously associated with a more migratory and invasive phenotype. This modulation is a key factor in the development of metastasis and disease progression and relapse. The promoted effects of cfDNA may result from its recognition through TLR9, inducing the activation of signaling pathways. The pro-tumoral potential of cfDNA for regulating the course of the disease will be influenced by the characteristics of the tumor microenvironment and the capability of cells to adapt.

Different subsets of cancer cells within a tumor behave differently, contributing in diverse ways for the disease to be successful and spread. Within the microenvironment, the cfDNA released by cancer cells may constitute a selective pressure to stimulate the evolution and selection of different subsets of cancer cells with different expertise. Here, we demonstrate that despite acting on the same processes, cfDNA variants that are isolated from cancer cells at different stages and culture conditions stimulate, in different ways, other cancer cells derived from the same cell line. Instant reactions were observed of adopting the metabolic profile that brings back the cell functioning of more favorable culture conditions that support proliferation. In the other hand, the long-term exposure to cfDNA induces quiescence that favors the chemoresistance of a subset of cancer cells. Therefore, different tumoral microenvironments may generate cfDNA variants that will impact cancer cells differently, orchestrating the disease’s fate (Figure 5). The cfDNA has a biological role in cancer, and more studies are needed to identify DNA sequences serving as markers for the development of tools for prevention, diagnosis, prognosis, and follow-up. While this study sheds light on the role of cfDNA in metabolic remodeling and chemoresistance in ovarian clear cell carcinoma, some limitations should be noted. Expanding the study to include more ovarian cancer cell lines would be crucial to evaluate the influence of heterogeneity on cfDNA release, to determine its role in cellular regulation, and to identify patterns of effects induced by cfDNA, as well as mechanisms specific to each subtype. The processes by which cfDNA alters metabolic pathways and chemoresistance are yet to be fully understood. Our findings show that TLR9 signaling plays a role, but more research utilizing inhibition techniques is needed to prove the pathway’s direct involvement. Furthermore, the cfDNA variations investigated here were obtained from a single cell line under particular culture conditions. Because the tumor microenvironment is extremely diverse, different cfDNA profiles may induce distinct cellular responses. Expanding this study to include cfDNA from various ovarian cancer subtypes and patient samples could have a greater clinical impact. Future research should seek to identify and validate certain cfDNA sequences that could be used as predictive or prognostic markers for disease progression and therapy response.

## Data Availability

Data is available in the public repository https://github.com/lgafeira/SelfDNA_MDA (accessed on 30 March 2025).
